# *In vitro* synergism of magnolol and honokiol in combination with antibacterial agents against clinical isolates of methicillin-resistant *Staphylococcus aureus* (MRSA)

**DOI:** 10.1186/s12906-015-0938-3

**Published:** 2015-12-01

**Authors:** Guo-Ying Zuo, Xin-Juan Zhang, Jun Han, Yu-Qing Li, Gen-Chun Wang

**Affiliations:** Research Center for Natural Medicines, Kunming General Hospital of Chengdu Military Command, Kunming, 650032 China; School of Pharmacy, Kunming Medical University, Kunming, 650032 China; School of Basic Medical Sciences, Yunnan Traditional Chinese Medical College, Kunming, 650500 China

**Keywords:** Magnolol, Honokiol, MRSA, Synergy, Antibacterial agent

## Abstract

**Background:**

Methicillin-resistant *Staphylococcus aureus* (MRSA) is a problematic pathogen posing a serious therapeutic challenge in the clinic. It is often multidrug-resistant (MDR) to conventional classes of antibacterial agents and there is an urgent need to develop new agents or strategies for treatment. Magnolol (ML) and honokiol (HL) are two naturally occurring diallylbiphenols which have been reported to show inhibition of MRSA. In this study their synergistic effects with antibacterial agents were further evaluated via checkerboard and time-kill assays.

**Methods:**

The susceptibility spectrum of clinical MRSA strains was tested by the disk diffusion method. The minimal inhibitory concentrations (MICs) and minimal bactericidal concentrations (MBCs) of ML and HL were assayed by broth microdilution. The synergy was evaluated through checkerboard microdilution and time-killing experiments.

**Results:**

ML and HL showed similar activity against both MSSA and MRSA with MIC/MBC at 16 ~ 64 mg/L, with potency similar to amikacin (AMK) and gentamicin (GEN). When they were used in combination with conventional antibacterial agents, they showed bacteriostatic synergy with FICIs between 0.25 ~ 0.5, leading to the combined MICs decreasing to as low as 1 ~ 2 and 1 ~ 16 mg/L for ML (HL) and the agents, respectively. MIC_50_ of the combinations decreased from 16 mg/L to 1 ~ 4 mg/L for ML (HL) and 8 ~ 128 mg/L to 2 ~ 64 mg/L for the antibacterial agents, which exhibited a broad spectrum of synergistic action with aminoglycosides (AMK, etilmicin (ETM) and GEN), floroquinolones (levofloxacin (LEV), ciprofloxacin and norfloxacin), fosfomycin (FOS) and piperacillin. The times of dilution (TOD, the extent of decreasing in MIC value) were determined up to 16 for the combined MIC. A more significant synergy after combining was determined as ML (HL) with AMK, ETM, GEN and FOS. ML (HL) combined with antibacterial agents did not show antagonistic effects on any of the ten MRSA strains. Reversal effects of MRSA resistance to AMK and GEN by ML and HL were also observed, respectively. All the combinations also showed better dynamic bactericidal activity against MRSA than any of single ML (HL) or the agents at 24 h incubation. The more significant synergy of combinations were determined as HL (ML) + ETM, HL + LEV and HL + AMK (GEN or FOS), with △LC_24_ of 2.02 ~ 2.25.

**Conclusion:**

ML and HL showed synergistic potentiation of antibacterial agents against clinical isolates of MRSA and warrant further pharmacological investigation.

## Background

The opportunistic pathogen *Staphylococcus aureus* (SA) is a leading cause of bacterial infections of people, causing a broad spectrum of pathology ranging from common skin infections to deep-seated fatal disease [1]. Antibiotic treatment of SA has once contributed greatly to human health for decades. However, due to long, wide and irrational applications of antibacterial agents in treatments in various fields other than in the clinic, methicillin-resistant *Staphylococcus aureus* (MRSA) has evolved as a problematic pathogen and has posed a serious therapeutic challenge in clinic [1, 2]. Nowadays, MRSA infections can be monitored both in hospitals [healthcare-acquired /associated (HA) MRSA] and community [community-acquired/associated (CA) MRSA]. The livestock-associated MRSA [(LA) MRSA] has also occurred [3]. MRSA is able to produce resistance to nearly all common classes of antibiotics including β-lactams, aminoglycosides, macrolides, tetracyclines and quinolones, and even the vancomycin-resistant *S. aureus* (VRSA) has also been reported [4]. The decreasing effectiveness of conventional drugs is continuously haunting both clinicians and drug researchers, and the critical shortage of new antibiotics in development against MRSA and other multidrug-resistant bacteria is of great concern worldwide. New targets with novel strategy of therapy and mechanism of action for development of antibacterial agents against MRSA are urgently needed [5].

Plant natural resources have been demonstrated to possess great chemical and biological diversities and promising findings of antibacterial phytochemicals which showed not only anti-MRSA activity alone, but also synergistic potentials when they were used in combination with conventional antibacterial agents against MRSA [6–10]. In recent years, we are devoting efforts to search for novel phytochemicals that showed synergy with conventional antibacterial agents against MRSA from medicinal plant sources, especially from the traditional Chinese medicines (TCM) [11–13]. We found two diallylbiphenols, *i.e.* magnolol (ML) and honokiol (HL) are such phytochemicals contained in the Chinese crude drug Hou-po, the stem or root bark of *Magnolia officinalis* Rehd. et Wils. (Magnoliaceae) [14].

Hou-po is an important species in TCM [14]. It has been traditionally used for respiration, digestion and infection related ailments like cough, diarrhea, and allergic rhinitis [14]. Modern pharmacological reports also demonstrated its antimicrobial, anti-inflammatory and analgesic, antianxiety and antidepressant, antitumor and anticoagulant effects as well as myocardial/cerebral ischemia protections [15]. ML and HL are two main phenolic constituents primarily isolated from Hou-po and also found in other Magnolia sp., together with other non-phenolic constituents such as alkaloids and essential oils [16–18]. The antimicrobial effects of Hou-po extracts on *Bacillus anthracis*, *S. aureus* and other pathogens were found as early as six decades ago [19–21]. Previous reports on antimicrobial activities of ML and HL and their synthetic derivatives include antibacterial [22–31], antifungal [32–34], antiviral [35] and nemicidal [36] activities. Although their antibacterial activities against MRSA and vancomycin-resistant enterococci (VRE) have been reported [37, 38], their potential for combined action on conventional antibacterial agents against MRSA has not been studied. Only the synergy of honokiol with fluconazole against clinical isolates of azole-resistant *Candida albicans* [39], and synergistic effect of lysozyme on bactericidal activity of magnolol and honokiol against a cariogenic bacterium of *Streptococcus mutans* OMZ 176 [40] including their potentiation of the antitumor agents [41–46] were reported. In this paper, we will show the potential synergistic effects of ML and HL in combination with conventional antibacterial agents against clinical MRSA strains through the checkerboard and time-kill curve methods.

## Methods

### Antimicrobial agents and disks

**Fig. 1:**
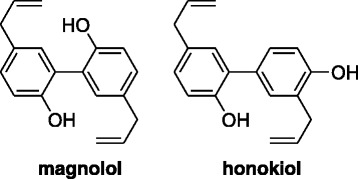
The structures of magnolol (ML) and honokiol (HL)

The eight antibacterial agents, i.e. amikacin (AMK) (Jiangsu Wuzhong Pharmaceutical Group Co., Ltd., Suzhou, China); Etilmicin (ETM) (Wuxi Jimin kexin Shanhe Pharmaceutical Co., Ltd.); gentamicin (GEN) (Guangzhou Baiyunshan Tianxin Pharmaceutical Co., Ltd., Guangzhou, China); Piperacillin (PIP) (Harbin Pharmaceutical Group Co., Ltd., Harbin, China); Norfloxacin (NOR) and Ciprofloxacin (CIP) (Sichuan Kelun Pharmaceutical Co., Ltd., Chengdu, China); Levofloxacin (LEV) (Yangzhijiang pharmaceutical Co., Ltd., Taizhou, China); Fosfomycin (FOS) (Northeast Pharmaceutical Group Co., Ltd., Shenyang, China). magnolol (ML) and honokiol (HL) (HPLC>98 %; Xian Xiaocao Science and Technology Co., Ltd., Xian, China) (Fig. 1). Vancomycin (VAN) (Eli Lilly Japan K. K., Seishin Laboratories) was used as the positive control agent. Cefoxitin (cfx, 0.03 mg) and other antibiotic impregnated disks were purchased from Beijing Tiantan biological products Co., Ltd., China.

### Bacterial strains and media

Ten MRSA strains (MRSA 01–10) were isolated and characterized from the infectious sputum sample of critically ill patients in KGH as previously reported [11–13]. The strains were determined with zone diameter (ZD) ≤ 21 mm against cefoxitin disk and the properties of susceptible (S), intermediate (I) and resistant (R) to antibacterial agents were determined according to the ZD Interpretive Criteria of Table 2C in 2012 CLSI by comparison with the ZD of corresponding antibacterial agents (Table 1) [47]. The presence of mecA gene and SCCmec genotypes were determined by multiplex PCR methods in Kunming Institute of Virology, PLA, China, as previously reported [48]. The control strain for MRSA was *S. aureus* (ATCC 25923; methicillin-susceptible *S. aureus* (MSSA)) which was purchased from the Beijing Tiantan Pharmaceutical and Biological Technology Co., Ltd., China. Standard Mueller-Hinton agar and broth (MHA and MHB, Tianhe Microbial Agents Co., Hangzhou, China) were used as bacterial culture media. Colony counts were determined using MHA plates. MHB was used for quantitative susceptibility testing and dynamic time–kill experiments.

**Table 1:** Resistance spectrum of the ten clinical isolates of MRSA strains

Strain	Resistant	Intermediate	Susceptible
MRSA 008	PEN, AMP, OXA, CFZ, Cfx, P/T, ERY, AZM, CIP, LEV, NOR, CLI		VAN, LZD, TEI
MRSA 082	PEN, AMP, OXA, CFZ, Cfx, FUR, CPZ/S, P/T, ERY, AZM, ClA, CIP, LEV, NOR, CLI, RIF	FOS	VAN, LZD, TEI
MRSA 098	PEN, AMP, OXA, CFZ, Cfx, FUR, CPZ/S, P/T, ERY, AZM, CIP, LEV, NOR, CLI, RIF		VAN, LZD, TEI
MRSA 111	PEN, AMP, OXA, CFZ, Cfx, FUR, CPZ/S, P/T, ERY, AZM, CIP, LEV, NOR, CLI, RIF		VAN, LZD, TEI
MRSA 135	PEN, AMP, OXA, CFZ, Cfx, FUR, CPZ/S, P/T, ERY, AZM, CIP, LEV, NOR, CLI, RIF		VAN, LZD, TEI, FOS
MRSA 144	PEN, AMP, OXA, CFZ, Cfx, FUR, CPZ/S, P/T, ERY, AZM, CIP, LEV, NOR, CLI, RIF		VAN, LZD, TEI
MRSA 166	PEN, AMP, OXA, CFZ, Cfx, FUR, CPZ/S, P/T, ERY, AZM, CIP, LEV, NOR, CLI, RIF, P/T		VAN, LZD, TEI, FOS
MRSA 187	PEN, AMP, OXA, CFZ, Cfx, FUR, CPZ/S, P/T, ERY, AZM, CIP, LEV, NOR, CLI, RIF		VAN, LZD, TEI, FOS
MRSA 189	PEN, AMP, OXA, CFZ, Cfx, FUR, CPZ/S, P/T, ERY, AZM, CIP, LEV, NOR, CLI, RIF		VAN, LZD, TEI, FOS
MRSA 321	PEN, AMP, OXA, CFZ, Cfx, FUR, P/T, ERY, AZM, CIP, LEV, NOR, CLI, CPZ/S, RIF	GAT, CTH, FOS	VAN

*PEN* Penicillin, *AMP* Ampicillin, *OXA* Oxacillin, *CFZ* Cefazolin, *Cfx* Cefoxitin, *FUR* Cefuroxime, *CTH* Cefathiamidine, C*PZ/S* Cefoperazone/sulbactam, *P/T* Piperacillin/tazobactam, *ERY* Erythromycin, *AZM* Azithromycin, *CIP* Ciprofloxacin, *GAT* Gatifloxacin, *LEV* Levofloxacin, *CLI* clindamycin, *RIF* Rifampicin, *CLA* Clarithromycin, *FOS* Fosfomycin, *LZD* Linezolid, *TEI* Teicoplanin, *VAN* Vancomycin

### Susceptibility testing

The test of susceptibility spectrum of the ten clinical MRSA strains to conventional antibacterial agents was performed by disk diffusion test following the CLSI guideline [47, 49, 50]. MICs/MBCs of ML and HL were determined by standardized broth microdilution techniques with inoculums of 5 × 10^5^ CFU/mL according to CLSI guidelines and incubated at 35 °C for 24 h [51–53]. The solvent used for dissolving the compounds and the antibiotics was MHB (or containing the final concentration of less than 5 % of dimethyl sulphoxide).

### Synergy testing

Potential interactions of ML and HL in combination with various antibiotics against MRSA were evaluated by determination of fractional inhibitory concentration indices (FICIs) and time-kill curves through using the checkerboard and dynamic time-kill methods as described previously [12, 13]. The bacteriostatic interaction mode was judged by FICIs as follows: FICI ≤ 0.5, synergy; 0.5 < FICI ≤ 1, additive; and 1 < FICI < 2, indifferent (or no effect) and FICI ≥ 2, antagonism [54, 55]. The bactericidal interaction mode was judged by the increased killing colony counts in log_10_ CFU/mL at 24 h incubation (△LC_24_) as follows: △LC_24_ ≥ 2 log_10_ CFU/mL, synergy; △LC_24_ = 1–2 log_10_ CFU/mL, additive; △LC_24_ = ±1 log_10_ CFU/mL, indifferent; △LC_24_ > −1 log_10_ CFU/mL, antagonism; where the △LC_24_ was calculated through the killing by a combination (LC_24_(co.)) deducting that by the most active single drug (LC_24_(si.)) in the combination, *i.e.* △LC_24_ = LC_24_(co.) - LC_24_(si.) [56].

### Statistical analysis

All the experiments were performed in triplicate. Data are expressed as the mean ± standard error. Statistical analyses were performed using the Statistical Package for the Social Sciences (SPSS 20.0) software (SPSS Inc., Chicago, IL, USA). Data were analysed by Kruskal–Wallis test and the significant differences between groups were analysed by Dunnett’s test. Statistical significance was accepted at a level of p < 0.01.

## Results

### Antimicrobial effects of ML and HL

The MICs/MBCs of ML, HL and eight conventional antibacterial agents alone against MSSA and MRSA are shown in Table 2. As a whole, ML and HL appeared as two moderate bactericidal agents against both MSSA and MRSA. The two compounds showed varied MIC/MBC of 16 ~ 64 mg/L against MSSA as most of the tested antibacterial agents, but they showed more potent activity than these agents against MRSA, with MIC_90_/MBC_90_ of 64/64 and 32/64 mg/L, respectively. HL showed the same activity as AMK, GEN and LEV with MIC/MBC of 16/32 mg/L against MSSA. The anti-MRSA potency of ML, HL and the eight agents followed the order of VAN > ETM > HL > ML > GEN > AMK > LEV (PIP, FOS) > CIP (NOR) judged by the values of MIC_90_/MBC_90_. Therefore, the antibacterial activity of the two compounds all showed more potent against MRSA than most of the conventional antibacterial agents with the exception of ETM and VAN (Table 2).

**Table 2:** MICs/MBCs (mg/L) of magnolol (ML), honokiol (HL) and conventional antibacterial agents against MSSA and the ten MRSA strains^a^

Agent^b^	MSSA	MRSA (*n* = 10)		
		Range	50%^c^	90%^d^
ML*	32/32	8 ~ 64*/16 ~ 128	16/16	64/64
HL*	16/32	16 ~ 32*/16 ~ 64	16/64	32/64
ETM	8/8	4 ~ 16/8 ~ 32	8/8	16/16
AMK	16/32	32 ~ 128*/64 ~ 256	64/128	64/256
GEN	16/32	16 ~ 128*/64 ~ 256	64/128	64/128
LEV	16/32	128 ~ 256/256 ~ 512	128/512	256/512
CIP	32/64	256 ~ 512/256 ~ 1024	256/512	512/1024
NOR	32/64	256 ~ 512/256 ~ 1024	256/512	512/1024
PIP	64/64	128 ~ 256/256 ~ 512	128/256	256/512
FOS	64/128	128 ~ 256/256 ~ 512	128/512	256/512
VAN	1/1	2/2	2/2	2/2

^a^ *MSSA* Methicillin-susceptible *Staphylococcus aureus* (ATCC25923), *MRSA* Methicillin-resistant *Staphylococcus aureus*

^b^ *ML* Magnolol, *HL* Honokiol, *AMK* Amikacin, *ETM* Etilmicin, *GEN* Gentamicin, *PIP* Piperacillin, *CIP* Ciprofloxacin, *LEV* Levofloxacin, *FOS* Fosfomycin, *NOR* Norfloxacin, *VAN* Vancomycin

^c^50 % = MIC_50_/MBC_50,_ *i.e.* the minimal inhibitory and bactericidal concentrations required to inhibit and kill 50 % of the strains, respectively

^b^90 % = MIC_90_/MBC_90_, *i.e.* the minimal inhibitory and bactericidal concentrations required to inhibit and kill 90 % of the strains, respectively

*p > 0.01

### Synergy of ML and HL in combination with antibacterial agents against MRSA

Table 3 shows the different degree of synergistic interactions of ML and HL in combination with the eight antibacterial agents against the ten clinical MRSA isolates. There are three to ten MRSA strains that showed synergy with FICIs between 0.25 ~ 0.5, leading to the combined MICs decreasing to as low as 1 ~ 2 and 1 ~ 16 mg/L for ML (HL) and the agents, respectively. MIC_50_ of the combinations decreased from 16 mg/L to 1 ~ 4 mg/L for ML (HL) and 8 ~ 128 mg/L to 2 ~ 64 mg/L for the antibacterial agents, which exhibited a broad spectrum of synergistic action with aminoglycosides (amikacin, etilmicin and gentamicin), floroquinolones (levofloxacin, ciprofloxacin and norfloxacin), fosfomycin and piperacillin. The times of dilution (TOD, the extent of decreasing in MIC value) were determined up to 16 for the combined MIC. The more significant synergy after combining was determined as ML (HL) with AMK, ETM, GEN and FOS. Therefore, ML and HL showed generally the same synergistic bacteriostatic effects on the tested antibacterial agents. Moreover, ML (HL) combined with antibacterial agents did not show antagonistic effects on any of the ten MRSA strains. There were only 1 ~ 2 strains that showed indifference.

**Table 3:** MICs (mg/L) of magnolol (ML) and honokiol (HL) used alone and in combination with antibacterial agents against the ten MRSA strains

Combination^a^	Effect	MIC (mg/L)			FICI^c^	Interaction (n)^d^		
		Alone	Combined	TOD^b^		Syn	Add	Ind
ML + AMK*	Range	8 + 32 ~ 32 + 128	2 + 4 ~ 8 + 32	8 + 16 ~ 2 + 2	0.375 ~ 0.75	8	2	0
	50 %	16 + 64	2 + 8	4 + 4	0.5			
	90 %	16 + 64	4 + 32	4 + 4	0.625			
ML + ETM	Range	8 + 4 ~ 32 + 16	1 + 1 ~ 8 + 4	16 + 8 ~ 2 + 4	0.313 ~ 0.75	8	2	0
	50 %	16 + 8	2 + 2	4 + 4	0.375			
	90 %	16 + 16	4 + 2	2 + 4	0.75			
ML + GEN*	Range	8 + 16 ~ 32 + 128	1 + 4 ~ 16 + 32	16 + 8 ~ 1 + 2	0.25 ~ 1.25	7	2	1
	50 %	16 + 64	2 + 8	8 + 4	0.5			
	90 %	16 + 64	4 + 32	2 + 2	1			
ML + FOS	Range	8 + 128 ~ 32 + 256	2 + 16 ~ 16 + 128	8 + 8 ~ 2 + 1	0.25 ~ 1.5	7	2	1
	50 %	16 + 128	4 + 32	4 + 8	0.375			
	90 %	16 + 256	8 + 64	2 + 4	0.75			
ML + CIP	Range	8 + 256 ~ 32 + 512	1 + 32 ~ 16 + 512	16 + 8 ~ 2 + 1	0.25 ~ 1.5	5	4	1
	50 %	16 + 256	4 + 64	4 + 8	0.5			
	90 %	16 + 512	8 + 256	2 + 2	0.75			
ML + LEV	Range	8 + 128 ~ 32 + 256	2 + 16 ~ 16 + 128	8 + 8 ~ 1 + 1	0.25 ~ 1.5	4	4	2
	50 %	16 + 128	4 + 32	4 + 8	0.625			
	90 %	16 + 256	8 + 128	2 + 2	1.25			
ML + NOR	Range	8 + 128 ~ 32 + 256	2 + 16 ~ 16 + 128	8 + 8 ~ 1 + 1	0.375 ~ 1.5	4	4	2
	50 %	16 + 128	4 + 64	4 + 4	0.625			
	90 %	16 + 256	8 + 128	2 + 2	1.25			
ML + PIP	Range	8 + 128 ~ 32 + 256	2 + 32 ~ 8 + 128	4 + 8 ~ 2 + 1	0.375 ~ 1.5	4	4	2
	50 %	16 + 128	4 + 32	4 + 4	0.75			
	90 %	16 + 256	8 + 128	2 + 1	1.25			
HL + ETM	Range	8 + 4 ~ 16 + 16	1 + 1 ~ 4 + 2	8 + 8 ~ 4 + 4	0.25 ~ 0.5	10	0	0
	50 %	16 + 8	2 + 2	8 + 4	0.375			
	90 %	16 + 16	4 + 2	4 + 4	0.5			
HL + AMK*	Range	8 + 32 ~ 16 + 128	1 + 4 ~ 4 + 32	8 + 8 ~ 4 + 2	0.25 ~ 0.75	9	1	0
	50 %	16 + 64	2 + 8	8 + 4	0.375			
	90 %	16 + 64	4 + 32	4 + 4	0.5			
HL + GEN*	Range	8 + 16 ~ 16 + 128	1 + 2 ~ 4 + 32	8 + 8 ~ 4 + 2	0.25 ~ 0.75	8	2	0
	50 %	16 + 64	2 + 16	8 + 4	0.375			
	90 %	16 + 64	4 + 32	4 + 2	0.625			
HL + FOS	Range	8 + 128 ~ 16 + 256	2 + 8 ~ 8 + 128	8 + 3 ~ 21 + 2	0.25 ~ 1.25	7	2	1
	50 %	16 + 128	4 + 32	4 + 4	0.5			
	90 %	16 + 256	8 + 64	2 + 2	1			
HL + CIP	Range	8 + 256 ~ 16 + 512	2 + 16 ~ 8 + 512	8 + 16 ~ 2 + 1	0.25 ~ 1.5	6	3	1
	50 %	16 + 256	2 + 64	4 + 4	0.5			
	90 %	16 + 512	8 + 256	2 + 2	0.75			
HL + LEV	Range	8 + 128 ~ 16 + 256	2 + 16 ~ 16 + 64	8 + 8 ~ 1 + 4	0.25 ~ 1.25	5	4	1
	50 %	16 + 128	4 + 32	4 + 8	0.5			
	90 %	16 + 256	8 + 32	2 + 4	1.125			
HL + PIP	Range	8 + 128 ~ 16 + 256	1 + 16 ~ 16 + 128	8 + 8 ~ 1 + 1	0.25 ~ 1.5	5	3	2
	50 %	16 + 128	2 + 32	8 + 4	0.5			
	90 %	16 + 256	8 + 128	2 + 2	1.25			
HL + NOR	Range	8 + 128 ~ 16 + 256	1 + 16 ~ 8 + 256	8 + 8 ~ 1 + 1	0.25 ~ 1.5	3	5	2
	50 %	16 + 128	4 + 64	4 + 4	0.75			
	90 %	16 + 256	8 + 128	2 + 2	1.25			

^a^ *ML* Magnolol, *HL* Honokiol, *AMK* Amikacin, *FOS* Fosfomycin, *LEV* Levofloxacin, *ETM* Etilmicin, *PIP* Piperacillin, *CIP* Ciprofloxacin, *NOR* Norfloxacin, All data on the left side of “+” belong to ML or HL, and the data on the right side of “+” belong to the conventional antibacterial agents, for example, “ML + AMK” means ML combined with AMK. ^b^ *TOD* Times of dilution = MIC_Alone_ /MIC_Combined_, ranged from the maximum to the minimum. ^c^ *FICI* Fractional inhibitory concentration index, ^d^ *Add* Additivity (0.5 < FICI ≤ 1), *Ind* Indifference (1 < FICI ≤ 2), *Syn* Synergy (FICI ≤ 0.5). *n’* Number of MRSA strains which showed the interactions. The total number is *n* = 10, e.g. *n* = n’(s) + n’(a) = 8 + 2 for ML + AMK combination in the first line in the table. *No statistically significant differences among the combinations of ML + AMK, ML + GEN, HL + AMK and HL + GEN (*p* >0.01)

**Fig. 2:**
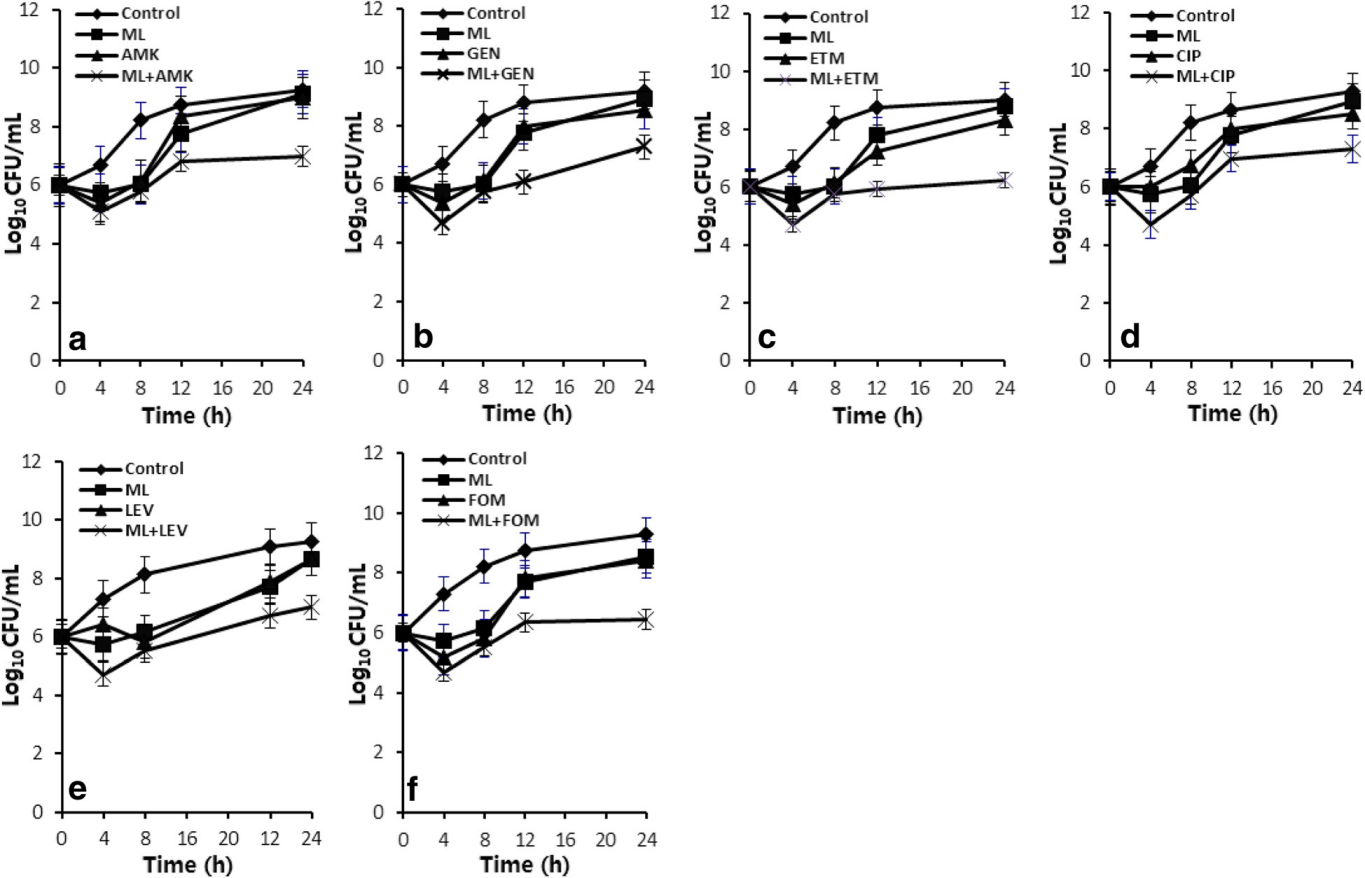
Time-killing curves of six combinations of ML with AMK (**a**), GEN (**b**), ETM (**c**), CIP (**d**), LEV (**e**) and FOS (**f**) at 1 × MIC and 24 h incubation against a representative clinical MRSA144 strain. Data are expressed as the mean ± standard error

**Fig. 3:**
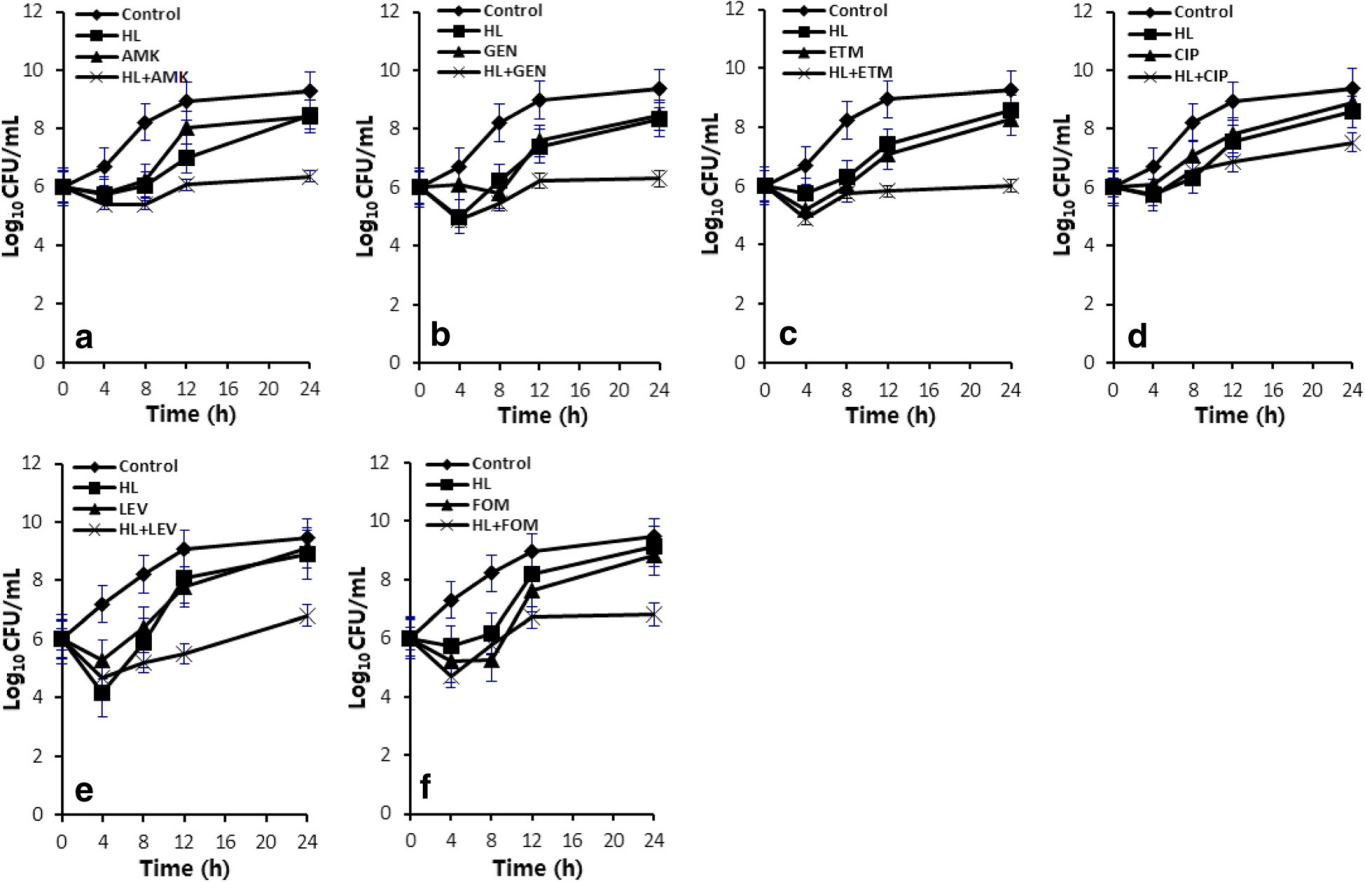
Time-killing curves of six combinations of HL with AMK (**a**), GEN (**b**), ETM (**c**), CIP (**d**), LEV (**e**) and FOS (**f**) at 1 × MIC and 24 h incubation against a representative clinical MRSA144 strain. Data are expressed as the mean ± standard error

To further evaluate the dynamic bactericidal effects of ML (HL) in combination with the antibacterial agents, the time-kill curve experiments were performed and the results are shown in Table 4 and Figs. 2 and 3. Eight of the combinations showed synergy in dynamic kill effects, with the order of potency as HL + ETM > HL + LEV >ML + ETM > HL + AMK > HL + FOS > HL + GEN > ML + AMK > ML + FOS. The rest of the four combinations ML + LEV, ML + CIP, ML + GEN and HL + CIP showed additive effects (Table 4). All the combinations showed better bactericidal activity against MRSA than any of single ML (HL) or the agents at 24 h incubation. The bactericidal efficiency of the combinations generally lasted longer than that of the single agents (Figs. 2 and 3). The more significant combinations were determined as HL + ETM, HL + LEV and ML + ETM, with △LC_24_ of 2.08 ~ 2.25 (Table 4). The combinations of antibacterial agents with HL showed more significant killing effects than those with ML for a same antibacterial agent. For example, the combination of HL + LEV showed synergy but the combination of ML + LEV showed only additively. Therefore, HL is a more optimistic agent for bactericidal potentiation of the effect of conventional antibacterial agents (Table 4).

**Table 4:** Collected time-killing assay results of various combinations of ML and HL with antibacterial agents at 24 h incubation against a clinical MRSA144 strain

Combination^a^	Masc^b^	△LC_24_(Int)^c^
HL + ETM	ETM	2.25 ± 0.12(Syn)*
HL + LEV	HL	2.09 ± 0.09(Syn)*
ML + ETM	ETM	2.08 ± 0.1(Syn)*
HL + AMK	AMK(~ML)	2.05 ± 0.07(Syn)*
HL + FOS	FOS	2.04 ± 0.03(Syn)
HL + GEN	HL	2.02 ± 0.01(Syn)
ML + AMK	AMK(~ML)	2.02 ± 0.02(Syn)
ML + FOS	FOS(~ML)	2.00 ± 0.01(Add)
ML + LEV	LEV(=ML)	1.64 ± 0.04(Add)
ML + CIP	CIP	1.24 ± 0.05(Add)
ML + GEN	GEN	1.24 ± 0.03(Add)
HL + CIP	HL	1.06 ± 0.04(Add)

^a^ *SAL* Salvianolate, *AMP* Ampicillin, *CAZ* Ceftazidime, *CFZ* Cefazolin, *CPS* Cefoperazone-sulbactam, *PTZ* Piperacillin-tazobactam, *AMK* Amikacin, *CLI* Clindamycin, *ERY* Erythromycin, *FOS* Fosfomycin, *LEV* Levofloxacin

^b^ *Masc* Most active single drug

^c^△*LC*_*24*_ △Log_10_CFU/mL at 24 h, *Int* Interaction, *Syn* Synergy (△LC_24_ ≥ 2), *Add* Additivity (1 < △LC_24_ < 2), *Ind* Indifference (△LC_24_ = ±1). Data are expressed as the mean ± standard error. *p <0.05

### Reversal of MRSA resistance to amikacin and gentamicin by ML and HL

**Fig. 4:**
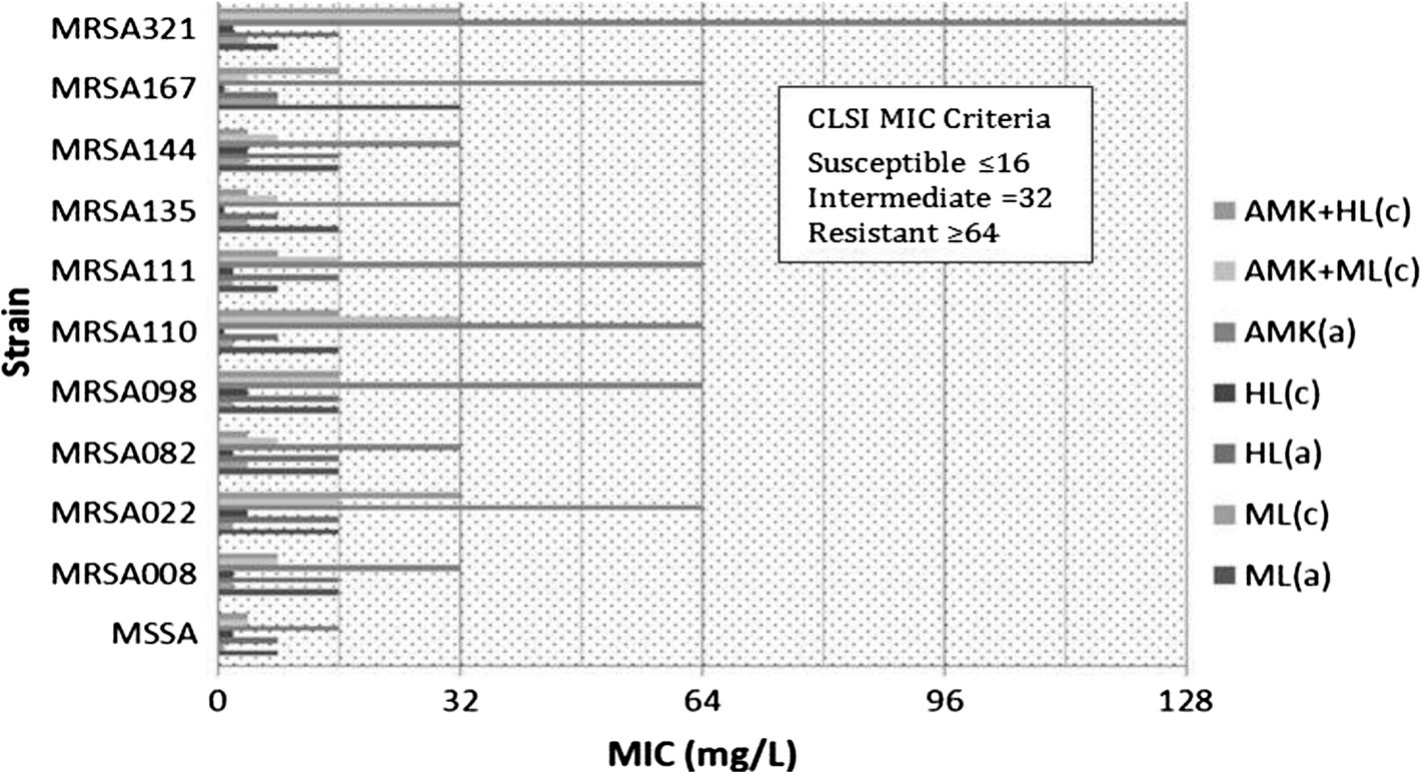
Reversal of MRSA resistance to AMK when it was used with combination with ML* and HL*, respectively (*p >0.01; a: alone; c: combined)

Besides the synergy effectiveness, the combination of ML (HL) with amikacin (AMK) led MICs (mg/L) of AMK to decrease markedly even to reverse the MRSA resistance to AMK by the MIC Interpretive Criteria of CLSI Performance Standards, *i.e.* MIC ≤16 mg/L (susceptible), MIC =32 mg/L (intermediate), MIC ≥64 mg/L (resistant) [47]. There is an equivalent eight strains of MRSA (*n* = 10) that showed MICs ≤16 mg/L against AMK when it was used in combination with ML and HL, respectively (*p* <0.01) (Table 3 and Fig. 4). Similarly, three strains of MRSA against GEN (MIC ≤4 mg/L (susceptible), MIC =8 mg/L (intermediate), MIC ≥16 mg/L (resistant)) also showed reversal interaction in combination with ML (HL) (Table 3) [47].

## Discussion

In the present report, we performed the antibacterial evaluations of ML and HL against MRSA both used alone and in combination with clinical conventional antibacterial agents of broad classes. The synergism of ML and HL on MRSA is found for the first time so far to the best of our knowledge [37, 39]. We determined the antibacterial activity of ML and HL against MRSA alone with MIC_50_/MBC_50_ (*n* = 10) of 16 ~ 64 mg/L which are similar to the results of the previous report which showed MIC/MBC of 12.5/25 mg/L [37]. The difference is reasonable for the varied MRSA strains used. From the results of combinatory effect on MRSA and the reported antifungal synergism [39], the two compounds are demonstrated to possess a wide spectrum of antimicrobial potentiation of conventional chemotherapy. It would be beneficial for the treatment of mixed infections and even the co-infection of tumor diseases, considering ML (HL) also potentiating antitumor agents [41–46].

It is important and valuable of ML and HL that potentiate the antimicrobial activity of aminoglycosides and other antibacterial drugs against MRSA, which could prevent the drugs from development of MDR to the troublesome germ. As aminoglycosides are one class of the important antimicrobials for the treatment of infectious diseases, the MRSA resistance reversal effects of ML and HL to the aminoglycosides agents (AMK, ETM and GEN) are especially significant for their application with largely reducing toxic reactions of the hosts’ liver, kidney and neural system by a substantially lowered dosage. Hence, greater sample scales are needed in order to draw a more reliable significance of the effectiveness on MRSA from clinical specimens and antibiotics.

The mechanism of the action of ML and HL against MRSA together with their synergism with antibacterial agents is still an unmet question. Two previous reviews summarized four main resistance mechanisms from bacteria: (i) receptor or active site modification, (ii) enzymatic degradation or modification of antibiotic, (iii) decreased penetration, or (iv) increased efflux [9, 10]. It was reported that the antimicrobial mechanisms of *Magnolia officinalis* extract resulted mainly in cell membrane and wall damage, causing increased permeability of cell membranes or lysis of cell walls and loss of cellular constituents, impairment of structural components and changes in bacterial cell morphology [21], which could ascribed to ML and HL, the two main constituents of the plant. Some studies also have demonstrated that increased permeability of the bacterial plasma membrane plays an important role in modulating resistance to aminoglycoside [57, 58]. Another study showed a phenolic diterpene totarol inhibits multidrug efflux pump activity in *Staphylococcus aureus* [59]. Therefore, the agents in the present report which shows the resistance reversal effects on the aminoglycosides (AMK and GEN) or synergistic potentiation of other conventional drugs could be through these mechanisms to a certain degree, though the real mechanism is remained to be clarified.

There are additional evidences revealing ML and HL as the modulators of the microbial membrane permeability. The two compounds showed active to extremely broad pathogenic microbial species. Apart from MSSA and MRSA, they showed as well antimicrobial activities against many other bacteria [19–31] and fungi [32–34] species and even exhibited antiviral [35] and nemicidal [36] activities. This antimicrobial mode is suggested like the antibacterial compounds of surface-active types which share the characters of usually nonselective to bacteria and very close MIC/MBC concentrations [30]. Therefore, ML and HL very likely target the extra cytoplasmic region as a nonionic surfactant and thus does not need to enter the cell, thereby avoiding most cellular pump-based resistance mechanisms as previously proposed [30]. The effects of ML (HL) on MRSA present here will expand the knowledge of their antimicrobial action and the future direction of anti-MDR investigations for drug development.

## Conclusions

ML and HL showed a broad spectrum of synergistic potentiation of conventional antibacterial agents, especially the resistance reversal of AMK and GEN against clinical MRSA isolates. These *in vitro* activities of ML and HL might partly ascribe to modulate the bacterial cell membrane penetration and warrant further pharmacological investigation.

## Ethics statement

The study was conducted in compliance with the ethics principles of the Declaration of Helsinki and Good Clinical Practice and China regulatory requirements. The study protocol (RCNM0116) was approved 10 June 2011 by the Ethics Committee and health authorities of Kunming General Hospital (KGH). Written informed consent was obtained from all subjects prior to sample commencement.
